# Approaching Alcohol Problems Through Local Environmental Interventions

**Published:** 2002

**Authors:** Andrew J. Treno, Juliet P. Lee

**Affiliations:** Andrew J. Treno, Ph.D., is a research scientist, and Juliet P. Lee, Ph.D., is an associate research scientist at the Prevention Research Center, Berkeley, California

**Keywords:** environmental-level prevention, community-based prevention, program evaluation, prevention outcome, model prevention strategy or program, prevention of problematic alcohol or other drug use (AODU), international differences, survey of research

## Abstract

One approach for reducing alcohol and other drug problems is community-based prevention programs. These programs focus on changing the environment in which a person consumes alcohol rather than the behavior of the individual drinker. Several international and U.S. programs have assessed the effectiveness of such approaches in reducing alcohol-related problems. Some of those analyses have had inconclusive results. Others, however, found reductions in alcohol-related problems such as drunk driving, alcohol-related car crashes and their consequences, the sale of alcohol to underage drinkers, and assault injuries. Nevertheless, several aspects of community-based prevention programs require further study.

For some chronic health problems, such as cardiovascular disease (CVD), community-based prevention programs have been effective in reducing those problems (Hulley and Fortmann 1981; see also Puska et al. 1985; Maccoby et al. 1977; Jacobs et al. 1986). Accordingly, researchers, community organizers, and funding agencies are examining the potential of community prevention programs for reducing alcohol and other drug (AOD)-related problems (Murray 1986). However, several important differences exist between programs aimed at reducing chronic health problems and those designed for addressing AOD problems. These differences concern philosophies and motivational strategies used in the programs and/or the characteristics of the target problems.

Comparing programs to reduce AOD and CVD as an example, the following four differences arise. First, interventions for high-risk medical conditions, such as changing dietary habits for CVD, operate under the assumption that people suffering from this condition have the power to control their behavior rationally. Conversely, efforts to reduce alcohol-related problems do not necessarily operate under this assumption.

Second, both greater needs and greater opportunities exist for regulating behaviors associated with alcohol-related problems than for regulating behaviors associated with CVD. For example, unlike poor dietary habits and smoking, which primarily affect the person exhibiting these behaviors (with the notable exception of secondhand smoke), alcohol consumption impacts the broader community system through traffic and other injuries. At the same time, alcohol consumption occurs within a highly regulatable distribution system of bars, restaurants, and other establishments.

Third, the consumption of alcohol often is more closely linked in time and space to the resulting alcohol-related problems (e.g., drunk driving and car crashes that occur shortly after drinking at a party) than are poor dietary habits (e.g., consumption of high-fat-content foods) and the resulting CVD, which may develop only after years of unhealthy eating. And fourth, societal norms associated with drinking differ dramatically from those associated with problematic dietary patterns. For example, because the decisions made by drinking drivers have consequences beyond the drinker him- or herself, the drinker’s behaviors are perceived as legitimate targets for social control and regulation.

Thus, although much may be learned from the experiences of CVD prevention programs, the specific methods that effectively reduce chronic health problems may be somewhat less applicable to the reduction of alcohol-related problems. The circumstances that surround alcohol consumption and the generation of alcohol-related problems may present unique challenges and strategic opportunities for the development of community prevention programs. In fact, with these differences in mind, researchers, community stakeholders, and funding agencies have increasingly turned to so-called environmental approaches for reducing alcohol-related problems. This article reviews current research on the effectiveness of such approaches. After briefly describing the theoretical framework of community-based environmental approaches, the article presents programs that have been implemented in the United States and in other countries and discusses future directions in the study of environmental approaches. The prevention projects included in this review were selected using criteria that, in the absence of random assignment to treatment and control conditions,^1^ represent the following scientifically minimal conditions for determining program efficacy:

- The studies include a careful collection of baseline data during the period preceding the intervention.
- They target well-defined community-level alcohol-related problems (e.g., trauma, alcohol-related violence, and initiation of drinking).
- They have a long-term implementation and monitoring period.
- They are followed by a final evaluation of changes in target problems.
- They involve empirically documented, successful or at least promising results in the target problem that are attributable to the intervention.

## Characterization of Community-Based Environmental Prevention Programs

In general, community-based environmental prevention programs as described here (subsequently referred to as environmental programs) focus not so much on changing the behavior of the individual drinker but on changing the environment in which a person consumes alcohol. The difference between environmental approaches and other approaches can best be clarified by considering the difference between “problem drinking” and “drinking-related problems.” The term “problem drinking” describes the behavior of an individual, and the treatment and prevention of problem drinking address the personal costs of drinking to the individual drinker, such as jeopardizing health, work, and family life. Conversely, “drinking-related problems” refers to consequences of alcohol consumption that affect many other people besides the individual drinker, including family, colleagues, neighbors, and other members of the community. These consequences include the health, social, and economic costs of drinking to the larger community, such as alcohol-related traffic crashes, alcohol-involved violence (e.g., assaults, homicides, rapes, and domestic abuse), and school dropout rates.

On the individual level, problem drinkers disproportionately contribute to drinking problems (i.e., each problem drinker generates more drinking-related problems than each nonproblem drinker). Nevertheless, because most people who consume alcohol cannot be classified as problem drinkers, the majority of drinking-related problems arise from nonproblem drinkers. As a result, community-based environmental prevention efforts seek to address these wider-scale problems caused by nonproblem drinkers rather than those caused by problem drinkers.

Because of this broader scope, environmental approaches to the prevention of alcohol-related problems operate differently than do individual treatment and prevention approaches. For example, environmental approaches focus on the community as a system involving numerous components, including the following:

- Individual drinkers
- Vendors of alcohol, including both places where alcohol is consumed (e.g., bars and restaurants) and places where alcohol is sold (e.g., liquor stores and other shops)
- Social events where alcoholic beverages are sold and consumed
- Local laws, regulations, and enforcement agencies
- Local medical clinics and treatment facilities
- Social organizations that may support and promote public health campaigns, including schools and PTAs, churches, business organizations, and social clubs.

Other, less obvious components of the community system affecting and affected by alcohol problems include the social networks and family connections through which people “learn” drinking attitudes and behaviors. These social networks may play a particularly important role with underage drinkers, who most likely obtain alcohol through friends and family networks (Harrison et al. 2000).

Although various applications of such a comprehensive environmental approach to the reduction of alcohol problems differ in terms of specific targets and tactics, they are all characterized by several strategies that distinguish them from more traditional information-based approaches that aim to change the alcohol use practices of individuals. First, environmental approaches use the media to target policymakers, as opposed to the general public, in support of program goals. Second, environmental approaches focus on mobilizing the community to make structural and system changes rather than on persuading individuals to change their behaviors. Third, environmental approaches generally target the supply of alcohol through social and market systems as opposed to the demand for alcohol by individuals (Gruenewald et al. in press). In sum, environmentally based community prevention programs represent an important and promising approach to the reduction of alcohol problems.

## Community-Based Environmental Intervention Outside the United States

Several interventions conducted outside the United States have provided useful guidelines for environmental prevention efforts. Together with efforts to evaluate so-called natural experiments,^2^ such as increases in the minimum legal drinking age (O’Malley and Wagenaar 1991; Hingson et al. 1994; Holder and Blose 1987), these interventions provide much of the scientific support for the design of current U.S. programs. The most salient of these international programs are the following:

- *The Community Action Project*, conducted during the early 1980s in six New Zealand communities, aimed to increase public support for alcohol regulation policies as well as induce changes in attitudes and behavior. The project involved a mass media campaign conducted in two communities, an intensive intervention media campaign and a community organizer in two other communities, and no intervention in two comparison communities (Casswell and Gilmore 1989). The project was designed to simultaneously effect change at the individual level by supporting healthy behaviors; at the community level by increasing support for policy change; and at the policy level by reducing both advertising and alcohol availability. Project outcomes, measured in a survey of respondents’ attitudes toward various alcohol control measures, included increased support for restrictions on alcohol sales in supermarkets as well as increased age limits in those communities that received the intensive intervention.
- *The Lahti Project*, conducted in Lahti, Finland, from 1992 to 1995, aimed to prevent alcohol-related problems through community action. The project included two intervention communities and two comparison communities. The interventions consisted of several modules: public educational activities conducted through media campaigns and community organizations; brief interventions in primary health care; media activities, community organizing, and training related to responsible beverage service; youth outreach and education utilizing school, community, and family channels as well as theater and video activities; and family counseling sessions (Holmila 1997). Although the project demonstrated efficacy in mobilizing community-level efforts, it was less effective in reaching the targeted program outcomes, such as achieving changes in public knowledge and perceptions of alcohol problems and changes in drinking and drinking-related problems. Thus, the investigators found no clear differences in drinking patterns or drinking-related problems that could be attributed to the project (Holmila 1995, 1997).
- *The Community Mobilization for the Prevention of Alcohol-Related Injury (COMPARI) project*, conducted in the Australian city of Geraldton between 1992 and 1995, was designed to reduce alcohol-related injuries by focusing not on heavy drinkers or alcoholics but on the general context of use in the community (Midford et al. 1999). Specific interventions included networking and support among community activists, community development, alternative options and health education, non-alcoholic youth activities, and policy interventions. However, the results of this project in terms of such outcomes as alcohol sales, assaults and traffic crashes, and hospital morbidity were inconclusive.

Despite their sometimes inconclusive results, these three international studies suggest that environmental approaches may be effective in reducing alcohol-related problems. At the same time, the ambiguous outcomes emphasize the importance of continued research in this area—for example, determining whether more intensive programs or different approaches would result in better outcomes.

## Community-Based Environmental Interventions in the United States

Three major U.S. environmental prevention projects have shown significant efficacy. These include the Saving Lives Project, Communities Mobilizing for Change on Alcohol (CMCA), and the Community Trials project. To illustrate the unique form and direction of environmentally based community prevention efforts, this section discusses these three projects in some detail.

### The Saving Lives Project

The Saving Lives Project, conducted in six Massachusetts communities with the rest of Massachusetts serving as a statistical control, aimed to reduce alcohol-impaired driving and related problems (Hingson et al. 1996). The specific programs implemented in each community were designed locally and involved such activities as media campaigns, business information programs, speeding and drunk-driving awareness days, speed-watch telephone hotlines, police training, high school peer-led education, Students Against Drunk Driving chapters, and college prevention programs. Over the 5 years of the program, the Saving Lives communities experienced a 33-percent decline in fatal crashes relative to the previous 5-year period. Furthermore, this decline was 42 percent greater than that observed in the rest of Massachusetts. Additionally there was a 47-percent reduction in the number of fatally injured drivers who tested positive for alcohol and a 39-percent decline in fatal crash injuries among 16- to 25-year olds relative to the rest of Massachusetts. Finally, self-reported driving after drinking declined in the experimental communities, particularly among youth (Hingson et al. 1996).

### The CMCA Project

This project, which included 15 communities in Minnesota and western Wisconsin, aimed to reduce access to alcohol among underage youth (Wagenaar et al. 2000*a*,*b*). The randomized trial was conducted in seven intervention communities, with eight communities serving as controls. The five core components of the project addressed the following issues: (1) community policies, (2) community practices, (3) youth alcohol access, (4) youth alcohol consumption, and (5) youth alcohol problems. Local organizers assisted community members in selecting interventions from an array of programs that affected youth access to alcohol. These measures included underage decoy operations with alcohol outlets, citizen monitoring of outlets selling to youth, keg registration, alcohol-free events for youth, policy action to shorten hours of sale for alcohol, responsible beverage service training programs, and educational programs for youth and adults. The experimental sites were free to modify and shape these intervention activities to address local conditions.

Analyses of the results of the project showed that merchants increased checks for age identification, reduced sales to minors, and reported more care in controlling sales to youth in the experimental communities (Wagenaar et al. 2000*b*). Attempts by underage youth to purchase alcohol as well as levels of alcohol use and the propensity to provide alcohol to other teens were reduced among the youth (Wagenaar et al. 2000*a*). Furthermore, the results demonstrated a decline in drinking-and-driving arrests among 18- to 20-year olds and disorderly conduct violations among 15- to 17-year olds (Wagenaar et al. 2000*b*).

### The Community Trials Project

The Community Trials Project (Holder et al. 1997) was a five-component community-level intervention conducted in three experimental communities that were matched with three comparison sites. The goal of the project, broadly defined, was to reduce alcohol-related harm among all residents of the three experimental communities. The project implemented the following five intervention components:

- *A media and mobilization component* to develop community organization and support for the goals and strategies of the project and to increase local news to raise public support for the goals and strategies of the project
- *A responsible beverage service component* to reduce service to intoxicated patrons at bars and restaurants
- *A sales to youth component* to reduce underage access to alcohol
- *A drinking and driving component* to increase local driving-while-intoxicated enforcement activity
- *An access component* to reduce the availability of alcohol.

Postintervention analysis demonstrated several important improvements in the experimental communities versus the comparison communities. For example, nighttime crashes resulting in injuries were 10 percent lower and crashes in which the driver was found by police to have been drinking were 6 percent lower in the experimental communities than in the comparison communities. Moreover, assault injuries observed in emergency departments declined by 43 percent, and assault injuries requiring hospitalization declined by 2 percent in the intervention communities versus the comparison communities. In addition, as indicated in a random-digit dialed telephone survey of community residents, self-reports of driving after having had “too much to drink” declined by 49 percent and self-reports of driving when “over the legal limit” declined by 51 percent in the experimental communities compared with the comparison communities. And although the drinking population increased slightly in the experimental communities over the course of the study, problematic alcohol use was significantly reduced in those communities (Holder et al. 2000). Finally, cost-benefit analyses estimated that the trial resulted in savings of $2.88 for every $1 spent on program implementation based upon reductions in automobile crashes alone (Holder et al. 1997). And this estimate is rather conservative because it does not take into consideration savings resulting from the prevention of other injuries, savings accruing over time after completion of the project as a result of the continuation of the intervention measures, or incalculable savings in terms of human suffering.

## Future Directions in Environmental Approaches

Although the studies reviewed above support the efficacy of community-based environmental prevention approaches, further research is needed to determine which aspects of this approach are the most promising or effective. Four key areas of such research are described below.

First, the relative efficacy of different preventive interventions must be assessed so that communities with limited resources can choose between alternative intervention strategies and select the most effective programs for their specific needs. To date, however, the efficacy of individual strategies must be inferred primarily from the programs’ effects on intermediary variables (e.g., reductions in alcohol access to youth or alcohol service to intoxicated patrons) because any distal effects (e.g. reductions in alcohol-related crashes or injuries) reflect the contribution of all interventions working in concert. For example, the combination of increased enforcement of laws against drinking and driving and increased coverage of that enforcement has been linked to increases in perceived risk of arrest for drinking and driving (i.e., the intermediary variable). This increased perception of risk has, in turn, been linked to decreases in drinking and driving and subsequent automobile crashes (i.e., the distal effect, which is also affected by other measures) (Voas et al. 1997).

Similarly, merchant training, enforcement, and media advocacy, when used in combination, can be effective in reducing underage purchases of alcohol (Grube 1997); training and enforcement would appear to be promising in terms of reducing service to intoxicated patrons (Saltz and Stanghetta 1997); and decreases in alcohol outlet densities have been linked to decreases in automobile crashes (Stockwell and Gruenewald 2001; Gruenewald et al. in press). All of these measures ultimately affect a distal variable, or major outcome of interest, such as alcohol-involved car crashes and the resulting injuries and deaths. These various pieces of information provide little conclusive information, however, on the individual contributions of these various interventions to changes in the outcome of interest.

Second, little information exists regarding the effectiveness of environmental programs from a cost-benefit perspective. For example, investigators still must determine the return on funds spent on such programs in terms of costs saved for treatment of alcohol-related injuries or lives saved. Communities facing the allocation of scarce resources will be very interested in the answers to such questions.

Third, the appropriate geographic focus of specific interventions remains unclear. Thus, future analyses must clarify whether interventions are best implemented at the community level or more locally, at the neighborhood level. One can argue that certain problems (e.g., drunk driving) are of a community-wide nature whereas others (e.g., drinking in public parks) are of a more local nature. The questions of who “owns” such problems or who is best equipped to address them are far from resolved.

Fourth, more research must be directed toward establishing the efficacy of local prevention programs in racial/ethnic minority neighborhoods, which typically experience different alcohol problems from those of non-minority communities. Toward this end, the National Institute on Alcohol Abuse and Alcoholism is currently sponsoring a project conducted by the Prevention Research Center, based in Berkeley, California, in two largely Hispanic, low-income neighborhoods in northern California to reduce under-age alcohol access, youth drinking, and related problems. This action project, to be conducted over a 5-year period, will include the following components:

- *A community awareness component* disseminating information about youth and young adult alcohol access and use
- *A responsible beverage service program* focusing on service to minors and intoxicated patrons at on- and off-sale establishments
- *An underage access component* providing support for increased police enforcement of underage sales laws and laws regarding provision of alcohol to minors by social hosts
- *An enforcement component* focusing on laws regulating sales to intoxicated people both in establishments and at special events where alcohol is served
- *A community mobilization effort* providing neighborhood support for the other components.

This project, though similar to the Community Trials project in terms of its environmental approach, differs from that project in three important regards. First, project interventions are to be implemented at the neighborhood level as opposed to the community level. Second, project interventions have been tailored to address the unique drinking problems and patterns characteristic of neighborhoods with large proportions of low-income ethnic minorities, such as high density of alcohol outlets and associated high levels of youth violence (Alaniz et al. 1998) and intensive alcohol advertising targeting ethnic minorities (Alaniz and Wilks 1998). Third, the focus of project interventions is on youth and young adults (ages 15 to 29), who disproportionately experience alcohol-related problems in these neighborhoods. With this approach, this project will address many of the areas noted above as requiring further exploration and should thereby help to establish the efficacy of community-based environmental interventions in preventing alcohol-related problems in a variety of settings.

## Footnotes

1 Although studies using random assignment would be ideal for determining treatment effectiveness, they are generally associated with prohibitive costs in the context of community prevention trials.

2 Natural experiments are those in which the outcomes of interventions that are outside the researchers’ control are evaluated.
